# 4-{[(4-Methyl­phen­yl)sulfon­yl]amino}­benzoic acid

**DOI:** 10.1107/S1600536811011524

**Published:** 2011-03-31

**Authors:** Ghulam Mustafa, Islam Ullah Khan, Muhammad Zia-ur-Rehman, Shahzad Sharif, Muhammad Nadeem Arshad

**Affiliations:** a Department of Chemistry, Government College University, Lahore 54000, Pakistan; b Applied Chemistry Research Centre, PCSIR Laboratories Complex, Lahore 54600, Pakistan

## Abstract

In the title compound, C_14_H_13_NO_4_S, the dihedral angle between the aromatic rings is 35.47 (10)°. In the crystal, adjacent mol­ecules are connected by pairs of O—H⋯O hydrogen bonds, forming head-to-head centrosymmetric dimers typical for carb­oxy­lic acids. Adjacent dimers are further linked through C—H⋯O inter­actions on one side and N—H⋯O inter­actions on the other, generating [010] chains.

## Related literature

For background to the biological activity of sulfonamides, see: Hanson *et al.* (1999 ▶). For related structures, see: Gowda *et al.* (2007 ▶); Arshad *et al.* (2008 ▶); Shafiq *et al.* (2009 ▶).
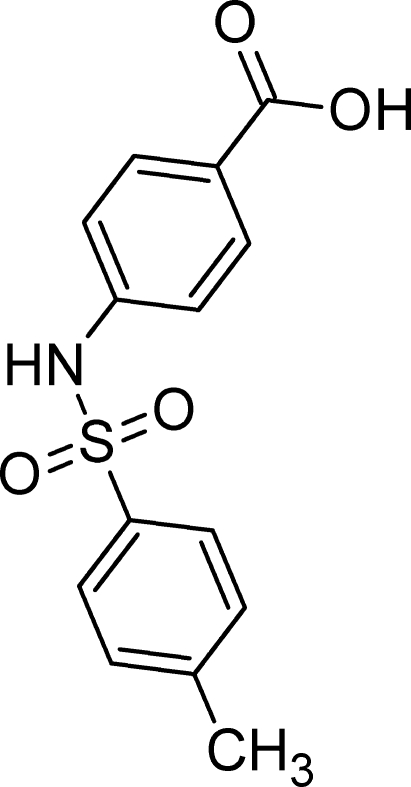


## Experimental

### 

#### Crystal data

C_14_H_13_NO_4_S
*M* *_r_* = 291.31
Triclinic, 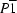

*a* = 5.1588 (2) Å
*b* = 6.9277 (2) Å
*c* = 20.0350 (6) Å
α = 83.574 (1)°
β = 86.357 (1)°
γ = 72.824 (1)°
*V* = 679.44 (4) Å^3^
*Z* = 2
Mo *K*α radiation
μ = 0.25 mm^−1^
*T* = 296 K
0.35 × 0.31 × 0.22 mm

#### Data collection

Bruker APEXII CCD diffractometer
12209 measured reflections
3313 independent reflections
2660 reflections with *I* > 2σ(*I*)
*R*_int_ = 0.029

#### Refinement

*R*[*F* ^2^ > 2σ(*F* ^2^)] = 0.044
*wR*(*F* ^2^) = 0.147
*S* = 1.01
3310 reflections
186 parameters
H atoms treated by a mixture of independent and constrained refinement
Δρ_max_ = 0.30 e Å^−3^
Δρ_min_ = −0.22 e Å^−3^

### 

Data collection: *APEX2* (Bruker, 2007 ▶); cell refinement: *SAINT* (Bruker, 2007 ▶); data reduction: *SAINT*; program(s) used to solve structure: *SHELXS97* (Sheldrick, 2008 ▶); program(s) used to refine structure: *SHELXL97* (Sheldrick, 2008 ▶); molecular graphics: *SHELXTL* (Sheldrick, 2008 ▶); software used to prepare material for publication: *SHELXTL*.

## Supplementary Material

Crystal structure: contains datablocks I, global, New_Global_Publ_Block. DOI: 10.1107/S1600536811011524/hb5824sup1.cif

Structure factors: contains datablocks I. DOI: 10.1107/S1600536811011524/hb5824Isup2.hkl

Additional supplementary materials:  crystallographic information; 3D view; checkCIF report

## Figures and Tables

**Table 1:** Hydrogen-bond geometry (Å, °)

*D*—H⋯*A*	*D*—H	H⋯*A*	*D*⋯*A*	*D*—H⋯*A*
N1—H1*N*⋯O1^i^	0.81 (3)	2.25 (3)	3.042 (2)	164.2 (2)
O3—H3*O*⋯O4^ii^	0.82	1.83	2.633 (2)	166
C5—H5⋯O3^iii^	0.93	2.55	3.397 (2)	151
C6—H6⋯O4^iv^	0.93	2.43	3.294 (3)	155

Symmetry codes: (i) 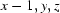; (ii) 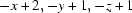; (iii) 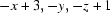; (iv) 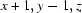.

**Fig. 1.**
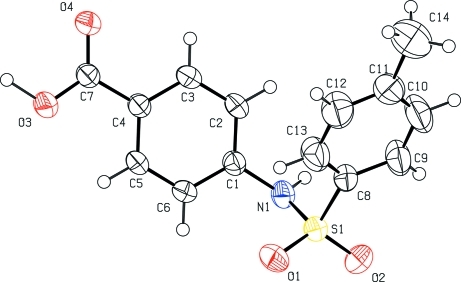
The moleuclar structure of (I) with displacement ellipsoids drawn at the 50% probability level.

**Fig. 2.**
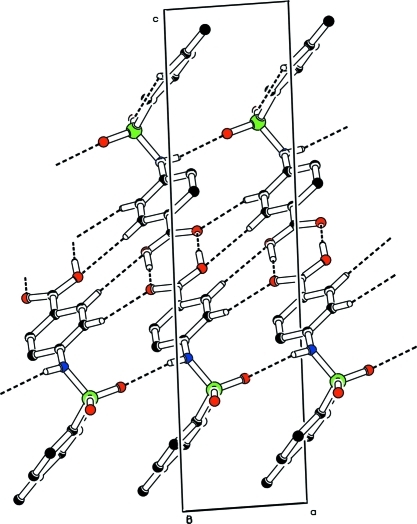
Unit cell packing diagram for (I) with hydrogen bonds shown as dashed lines.

## supplementary crystallographic information

### Comment

Sulfonamides are well known for their various types of biological activities (e.g. Hanson *et al.*, 1999). In the present paper, the structure of the title compound, (I), is reported. Bond lengths and bond angles of the title molecule are similar to those of the related molecules (Gowda *et al.*, 2007; Arshad *et al.*, 2008; Shafiq *et al.*, 2009) and are within normal ranges. In the crystal, each molecule is linked to its adjacent one through head-to-head pairs of O—H···O inter molecular interactions giving rise to dimeric motifs typical for carboxylic acids. Neighbouring dimers are further linked to each other through C—H···O interactions on one side and through N—H···O on the other side along *b* axis (Fig. 2).

### Experimental

To a mixture of *p*-amino benzoic acid (1.0 g, 7.3 mmoles) and distilled water (10 ml) in a round bottomflask (25 ml) 1*M* aqueous sodium carbonate solution was added to maintain the pH between 8–9. Tosyl chloride (1.66 g, 8.70 mmol) was added to this solution and was kept stirred at room temperature untill the suspension changed to a clear solution. pH of the reaction mixture was adjusted to 1–2, using 1 N HCl and the precipitates obtained were filtered, washed with distilled water, dried and recrystallized from methanol to yield colourless needles of (I).

### Refinement

The aromatic C—H H-atoms were positioned with idealized geometry with C—H = 0.93 Å and were refined using a riding model with *U*_iso_(H) = 1.2 *U*_eq_(C). The O—H H-atom was also positioned with idealized geometry with O—H = 0.82 Å and was refined using a riding model with *U*_iso_(H) = 1.5 *U*_eq_(O). The N—H H atom were located in difference map and its position refiined freely to N—H= 0.82 (2) Å, *U*_iso_(H) = 1.2U_iso_(N). Three reflection 011, 001 and 002 were omitted in the final refinement as these were obscured by beam stop.

### Crystal data

**Table:**

C_14_H_13_NO_4_S	*Z* = 2
*M**_r_* = 291.31	*F*(000) = 304
Triclinic, *P*1	*D*_x_ = 1.424 Mg m^−^^3^
Hall symbol: -P 1	Melting point: 503 K
*a* = 5.1588 (2) Å	Mo *K*α radiation, λ = 0.71073 Å
*b* = 6.9277 (2) Å	Cell parameters from 5143 reflections
*c* = 20.0350 (6) Å	θ = 3.1–27.8°
α = 83.574 (1)°	µ = 0.25 mm^−^^1^
β = 86.357 (1)°	*T* = 296 K
γ = 72.824 (1)°	Needles, colourless
*V* = 679.44 (4) Å^3^	0.35 × 0.31 × 0.22 mm

### Data collection

**Table:**

Bruker APEXII CCD diffractometer	2660 reflections with *I* > 2σ(*I*)
Radiation source: fine-focus sealed tube	*R*_int_ = 0.029
graphite	θ_max_ = 28.3°, θ_min_ = 1.0°
φ and ω scans	*h* = −6→6
12209 measured reflections	*k* = −9→9
3313 independent reflections	*l* = −26→26

### Refinement

**Table:**

Refinement on *F*^2^	Primary atom site location: structure-invariant direct methods
Least-squares matrix: full	Secondary atom site location: difference Fourier map
*R*[*F*^2^ > 2σ(*F*^2^)] = 0.044	Hydrogen site location: inferred from neighbouring sites
*wR*(*F*^2^) = 0.147	H atoms treated by a mixture of independent and constrained refinement
*S* = 1.00	*w* = 1/[σ^2^(*F*_o_^2^) + (0.0982*P*)^2^ + 0.0842*P*] where *P* = (*F*_o_^2^ + 2*F*_c_^2^)/3
3310 reflections	(Δ/σ)_max_ = 0.001
186 parameters	Δρ_max_ = 0.30 e Å^−^^3^
0 restraints	Δρ_min_ = −0.22 e Å^−^^3^

### Special details

**Table:**

Geometry. All s.u.'s (except the s.u. in the dihedral angle between two l.s. planes) are estimated using the full covariance matrix. The cell s.u.'s are taken into account individually in the estimation of s.u.'s in distances, angles and torsion angles; correlations between s.u.'s in cell parameters are only used when they are defined by crystal symmetry. An approximate (isotropic) treatment of cell s.u.'s is used for estimating s.u.'s involving l.s. planes.
Refinement. Refinement of *F*^2^ against ALL reflections. The weighted *R*-factor *wR* and goodness of fit *S* are based on *F*^2^, conventional *R*-factors *R* are based on *F*, with *F* set to zero for negative *F*^2^. The threshold expression of *F*^2^ > 2σ(*F*^2^) is used only for calculating *R*-factors(gt) *etc*. and is not relevant to the choice of reflections for refinement. *R*-factors based on *F*^2^ are statistically about twice as large as those based on *F*, and *R*- factors based on ALL data will be even larger.

### Fractional atomic coordinates and isotropic or equivalent isotropic displacement parameters (Å^2^)

**Table:**

	*x*	*y*	*z*	*U*_iso_*/*U*_eq_	
C1	1.0578 (3)	−0.2205 (2)	0.33980 (8)	0.0363 (3)	
C2	0.8392 (3)	−0.0474 (3)	0.33116 (9)	0.0450 (4)	
H2	0.7040	−0.0414	0.3017	0.054*	
C3	0.8230 (3)	0.1167 (3)	0.36655 (9)	0.0447 (4)	
H3	0.6746	0.2320	0.3617	0.054*	
C4	1.0281 (3)	0.1089 (2)	0.40922 (8)	0.0357 (3)	
C5	1.2453 (3)	−0.0655 (3)	0.41777 (8)	0.0406 (4)	
H5	1.3824	−0.0711	0.4465	0.049*	
C6	1.2585 (3)	−0.2306 (3)	0.38368 (9)	0.0415 (4)	
H6	1.4022	−0.3485	0.3902	0.050*	
C7	1.0157 (3)	0.2840 (2)	0.44689 (8)	0.0381 (4)	
C8	1.1343 (4)	−0.2279 (3)	0.17695 (9)	0.0435 (4)	
C9	0.9505 (5)	−0.2551 (3)	0.13444 (11)	0.0634 (6)	
H9	0.9213	−0.3814	0.1345	0.076*	
C10	0.8108 (6)	−0.0924 (4)	0.09195 (13)	0.0786 (7)	
H10	0.6877	−0.1112	0.0631	0.094*	
C11	0.8470 (5)	0.0958 (4)	0.09073 (12)	0.0670 (6)	
C12	1.0313 (6)	0.1180 (4)	0.13297 (14)	0.0757 (7)	
H12	1.0603	0.2444	0.1326	0.091*	
C13	1.1759 (5)	−0.0405 (3)	0.17613 (12)	0.0652 (6)	
H13	1.3002	−0.0211	0.2044	0.078*	
C14	0.6831 (8)	0.2757 (5)	0.04587 (16)	0.1065 (11)	
H14A	0.6420	0.2297	0.0055	0.160*	
H14B	0.7864	0.3703	0.0347	0.160*	
H14C	0.5172	0.3411	0.0691	0.160*	
N1	1.0818 (3)	−0.3933 (2)	0.30382 (7)	0.0424 (3)	
O1	1.5356 (2)	−0.3963 (2)	0.25576 (7)	0.0559 (4)	
O2	1.2874 (3)	−0.6138 (2)	0.21531 (8)	0.0617 (4)	
O3	1.2221 (3)	0.2721 (2)	0.47994 (7)	0.0549 (4)	
H3O	1.1959	0.3755	0.4989	0.082*	
O4	0.8069 (3)	0.43283 (18)	0.44517 (7)	0.0507 (3)	
S1	1.28689 (8)	−0.42565 (6)	0.23758 (2)	0.04325 (17)	
H1N	0.937 (4)	−0.412 (3)	0.2967 (10)	0.052*	

### Atomic displacement parameters (Å^2^)

**Table:**

	*U*^11^	*U*^22^	*U*^33^	*U*^12^	*U*^13^	*U*^23^
C1	0.0353 (8)	0.0366 (8)	0.0385 (8)	−0.0115 (6)	−0.0060 (6)	−0.0040 (6)
C2	0.0334 (8)	0.0505 (10)	0.0511 (10)	−0.0074 (7)	−0.0150 (7)	−0.0102 (8)
C3	0.0359 (8)	0.0417 (9)	0.0527 (10)	−0.0019 (7)	−0.0148 (7)	−0.0066 (7)
C4	0.0349 (8)	0.0357 (8)	0.0363 (8)	−0.0092 (6)	−0.0071 (6)	−0.0023 (6)
C5	0.0350 (8)	0.0420 (9)	0.0434 (9)	−0.0058 (7)	−0.0146 (6)	−0.0063 (7)
C6	0.0374 (8)	0.0368 (8)	0.0459 (9)	−0.0015 (7)	−0.0125 (7)	−0.0052 (7)
C7	0.0373 (8)	0.0374 (8)	0.0392 (8)	−0.0092 (6)	−0.0081 (6)	−0.0022 (6)
C8	0.0424 (9)	0.0475 (10)	0.0422 (9)	−0.0129 (7)	−0.0053 (7)	−0.0104 (7)
C9	0.0781 (14)	0.0604 (13)	0.0591 (12)	−0.0270 (11)	−0.0262 (11)	−0.0055 (10)
C10	0.0892 (17)	0.0836 (17)	0.0660 (14)	−0.0247 (14)	−0.0404 (13)	0.0000 (12)
C11	0.0753 (15)	0.0612 (13)	0.0551 (12)	−0.0048 (11)	−0.0143 (11)	−0.0003 (10)
C12	0.1027 (19)	0.0499 (13)	0.0779 (16)	−0.0253 (12)	−0.0269 (14)	0.0012 (11)
C13	0.0770 (15)	0.0537 (12)	0.0718 (14)	−0.0258 (11)	−0.0268 (11)	−0.0027 (10)
C14	0.124 (3)	0.089 (2)	0.088 (2)	−0.0063 (18)	−0.037 (2)	0.0206 (16)
N1	0.0406 (8)	0.0443 (8)	0.0478 (8)	−0.0178 (6)	−0.0080 (6)	−0.0087 (6)
O1	0.0349 (7)	0.0683 (9)	0.0644 (8)	−0.0120 (6)	−0.0093 (6)	−0.0104 (7)
O2	0.0688 (9)	0.0442 (8)	0.0713 (9)	−0.0070 (7)	−0.0126 (7)	−0.0218 (7)
O3	0.0473 (7)	0.0476 (8)	0.0707 (9)	−0.0054 (6)	−0.0238 (6)	−0.0202 (6)
O4	0.0473 (7)	0.0399 (7)	0.0603 (8)	0.0001 (5)	−0.0193 (6)	−0.0110 (6)
S1	0.0381 (3)	0.0422 (3)	0.0497 (3)	−0.00771 (18)	−0.00885 (18)	−0.01246 (18)

### Geometric parameters (Å, °)

**Table:**

C1—C6	1.382 (2)	C9—C10	1.378 (3)
C1—C2	1.386 (2)	C9—H9	0.9300
C1—N1	1.436 (2)	C10—C11	1.368 (3)
C2—C3	1.385 (2)	C10—H10	0.9300
C2—H2	0.9300	C11—C12	1.364 (3)
C3—C4	1.387 (2)	C11—C14	1.517 (3)
C3—H3	0.9300	C12—C13	1.377 (3)
C4—C5	1.387 (2)	C12—H12	0.9300
C4—C7	1.482 (2)	C13—H13	0.9300
C5—C6	1.379 (2)	C14—H14A	0.9600
C5—H5	0.9300	C14—H14B	0.9600
C6—H6	0.9300	C14—H14C	0.9600
C7—O4	1.251 (2)	N1—S1	1.6366 (16)
C7—O3	1.2670 (19)	N1—H1N	0.82 (2)
C8—C13	1.375 (3)	O1—S1	1.4321 (13)
C8—C9	1.380 (2)	O2—S1	1.4232 (13)
C8—S1	1.7564 (19)	O3—H3O	0.8200
			
C6—C1—C2	120.40 (15)	C11—C10—H10	118.9
C6—C1—N1	118.37 (14)	C9—C10—H10	118.9
C2—C1—N1	121.23 (14)	C12—C11—C10	117.6 (2)
C3—C2—C1	119.66 (15)	C12—C11—C14	120.8 (2)
C3—C2—H2	120.2	C10—C11—C14	121.7 (2)
C1—C2—H2	120.2	C11—C12—C13	122.2 (2)
C2—C3—C4	120.00 (15)	C11—C12—H12	118.9
C2—C3—H3	120.0	C13—C12—H12	118.9
C4—C3—H3	120.0	C8—C13—C12	119.23 (19)
C5—C4—C3	119.86 (14)	C8—C13—H13	120.4
C5—C4—C7	119.40 (14)	C12—C13—H13	120.4
C3—C4—C7	120.73 (14)	C11—C14—H14A	109.5
C6—C5—C4	120.15 (14)	C11—C14—H14B	109.5
C6—C5—H5	119.9	H14A—C14—H14B	109.5
C4—C5—H5	119.9	C11—C14—H14C	109.5
C5—C6—C1	119.89 (15)	H14A—C14—H14C	109.5
C5—C6—H6	120.1	H14B—C14—H14C	109.5
C1—C6—H6	120.1	C1—N1—S1	118.23 (11)
O4—C7—O3	123.49 (15)	C1—N1—H1N	114.4 (15)
O4—C7—C4	119.73 (14)	S1—N1—H1N	111.3 (15)
O3—C7—C4	116.78 (14)	C7—O3—H3O	109.5
C13—C8—C9	119.84 (19)	O2—S1—O1	119.96 (9)
C13—C8—S1	120.34 (14)	O2—S1—N1	106.32 (8)
C9—C8—S1	119.49 (15)	O1—S1—N1	106.96 (8)
C10—C9—C8	119.0 (2)	O2—S1—C8	108.93 (8)
C10—C9—H9	120.5	O1—S1—C8	108.33 (9)
C8—C9—H9	120.5	N1—S1—C8	105.41 (8)
C11—C10—C9	122.2 (2)		
			
C6—C1—C2—C3	0.3 (3)	C9—C10—C11—C14	−177.3 (3)
N1—C1—C2—C3	−179.80 (15)	C10—C11—C12—C13	−0.7 (4)
C1—C2—C3—C4	1.5 (3)	C14—C11—C12—C13	177.5 (3)
C2—C3—C4—C5	−1.8 (3)	C9—C8—C13—C12	0.4 (3)
C2—C3—C4—C7	179.29 (16)	S1—C8—C13—C12	−173.0 (2)
C3—C4—C5—C6	0.4 (3)	C11—C12—C13—C8	0.1 (4)
C7—C4—C5—C6	179.27 (15)	C6—C1—N1—S1	−79.40 (18)
C4—C5—C6—C1	1.4 (3)	C2—C1—N1—S1	100.69 (17)
C2—C1—C6—C5	−1.7 (3)	C1—N1—S1—O2	175.57 (12)
N1—C1—C6—C5	178.34 (15)	C1—N1—S1—O1	46.27 (15)
C5—C4—C7—O4	−172.22 (16)	C1—N1—S1—C8	−68.88 (13)
C3—C4—C7—O4	6.7 (3)	C13—C8—S1—O2	−161.30 (17)
C5—C4—C7—O3	7.4 (2)	C9—C8—S1—O2	25.26 (19)
C3—C4—C7—O3	−173.74 (16)	C13—C8—S1—O1	−29.26 (19)
C13—C8—C9—C10	−0.2 (3)	C9—C8—S1—O1	157.30 (17)
S1—C8—C9—C10	173.2 (2)	C13—C8—S1—N1	84.95 (18)
C8—C9—C10—C11	−0.4 (4)	C9—C8—S1—N1	−88.49 (18)
C9—C10—C11—C12	0.8 (4)		

### Hydrogen-bond geometry (Å, °)

**Table:**

*D*—H···*A*	*D*—H	H···*A*	*D*···*A*	*D*—H···*A*
N1—H1N···O1^i^	0.81 (3)	2.25 (3)	3.042 (2)	164.2 (2)
O3—H3O···O4^ii^	0.82	1.83	2.633 (2)	166
C5—H5···O3^iii^	0.93	2.55	3.397 (2)	151
C6—H6···O4^iv^	0.93	2.43	3.294 (3)	155

Symmetry codes: (i) *x*−1, *y*, *z*; (ii) −*x*+2, −*y*+1, −*z*+1; (iii) −*x*+3, −*y*, −*z*+1; (iv) *x*+1, *y*−1, *z*.
